# The Role of Cytokines in the Pathogenesis of Schizophrenia

**DOI:** 10.3390/jcm10173849

**Published:** 2021-08-27

**Authors:** Bartosz Dawidowski, Adrianna Górniak, Piotr Podwalski, Zofia Lebiecka, Błażej Misiak, Jerzy Samochowiec

**Affiliations:** 1 Department of Psychiatry, Pomeranian Medical University, 71-460 Szczecin, Poland; aravial.mg@gmail.com (B.D.); adrianna.gorniak@vp.pl (A.G.); samoj@pum.edu.pl (J.S.); 2 Department of Health Psychology, Pomeranian Medical University, 71-210 Szczecin, Poland; zofia.lebiecka@gmail.com; 3 Department of Psychiatry, Division of Consultation Psychiatry and Neuroscience, Medical University, 50-367 Wroclaw, Poland; blazej.misiak@umed.wroc.pl

**Keywords:** schizophrenia, cytokines, antipsychotics, psychosis, neuroinflammation

## Abstract

Schizophrenia is a chronic mental illness of unknown etiology. A growing and compelling body of evidence implicates immunologic dysfunction as the key element in its pathomechanism. Cytokines, whose altered levels have been increasingly reported in various patient populations, are the major mediators involved in the coordination of the immune system. The available literature reports both elevated levels of proinflammatory as well as reduced levels of anti-inflammatory cytokines, and their effects on clinical status and neuroimaging changes. There is evidence of at least a partial genetic basis for the association between cytokine alterations and schizophrenia. Two other factors implicated in its development include early childhood trauma and disturbances in the gut microbiome. Moreover, its various subtypes, characterized by individual symptom severity and course, such as deficit schizophrenia, seem to differ in terms of changes in peripheral cytokine levels. While the use of a systematic review methodology could be difficult due to the breadth and diversity of the issues covered in this review, the applied narrative approach allows for a more holistic presentation. The aim of this narrative review was to present up-to-date evidence on cytokine dysregulation in schizophrenia, its effect on the psychopathological presentation, and links with antipsychotic medication. We also attempted to summarize its postulated underpinnings, including early childhood trauma and gut microbiome disturbances, and propose trait and state markers of schizophrenia.

## 1. Introduction

Schizophrenia is a chronic mental disorder with a complex etiopathogenesis, which involves both congenital and environmental factors [1]. It leads to neurodegenerative changes in the central nervous system (CNS) and a significant impairment of social functioning [2,3,4]. Its lifetime incidence has been estimated at 7.1 per 1000 people, and the male to female risk ratio is 1.4:1 [5]. Alongside other, less pronounced symptoms, schizophrenia involves the presence of positive (e.g., delusions, hallucinations), negative (e.g., anhedonia, social withdrawal), and affective symptoms, as well as cognitive dysfunction (e.g., impaired working memory) [1].

Recent research indicates that subclinical inflammation in the CNS and immune dysregulation may play a role in the etiopathogenesis of schizophrenia, which is supported by immunogenetic evidence and a higher incidence of autoimmune diseases in patients with schizophrenia relative to the general population [6,7,8]. Neuroinflammation can lead to white matter pathology, dysconnectivity, and thus to the onset of schizophrenia symptoms [8].

Cytokines are signaling proteins that affect mainly immune cells, regulating their proliferation and activation [9]. Produced by a wide variety of cell populations, they play a critical role in the coordination of the inflammatory response [9]. Another factor considered important in studies on the etiopathogenesis of schizophrenia are chemokines, i.e., a subgroup of cytokines whose main role is to attract immune cells to the site of inflammation [10]. In addition, both chemokines and non-chemokine cytokines modulate the processes of neurogenesis and neural pruning, and may affect behavior [10,11].

Numerous studies, including many meta-analyses, demonstrate alterations in blood cytokine levels in schizophrenia patients compared to healthy controls (HC) [12,13,14,15,16]. Additionally, they tend to manifest the increased mRNA expression of cytokine genes in lymphocytes relative to HC [17]. This may stem from epigenetic mechanisms underlying the relationship between schizophrenia and stress in early childhood [18,19]. A known risk factor for schizophrenia, early childhood trauma is associated with poorer responses to treatment and symptom characteristics [18,20,21,22,23]. Elevated peripheral and cerebrospinal fluid (CSF) cytokine levels are hypothesized to partially result from disturbed gut microbiome composition, which were observed in patients with schizophrenia and may be caused by both maternal and developmental stress [24,25,26,27,28].

Schizophrenia research is typically conducted on a small patient population. The most extensively researched cohort is patients with first-episode psychosis (FEP), whose functioning is monitored prior to the initiation of antipsychotic treatment (first episode antipsychotics naive, FEAN). The second group consists of patients showing subsequent psychotic episodes, who receive treatment due to relapse (acute relapsed chronic, ARCh), while the third includes those in remission (stable chronic, SCh). Some other, far less studied patient populations include those with early onset psychosis (EOP) and patients at clinical high risk (CHR) or ultra-high risk (UHR) of psychosis.

The aim of this narrative review is to present the most valuable evidence on cytokine dysregulation in schizophrenia, the links between cytokine levels and psychopathological presentation, as well as their alterations in response to antipsychotics. We will also investigate the possible underpinnings of changes in the cytokine system.

## 2. Materials and Methods

Due to the large variety within existing research, as well as the immense amount of data accumulated over the years, including systematic reviews and meta-analyses, we decided against the use of systematic methodologies and instead resolved to present a narrative review. This strategy, despite certain limitations, enables a more holistic approach to the role of cytokine alterations in the etiopathogenesis of schizophrenia, which may prove particularly useful for clinicians and researchers alike who want to explore this very timely subject.

To this end, a PubMed literature search was conducted to identify all relevant studies and meta-analyses published between January 1st 2010 and April 22th 2021, using either “cytokines and schizophrenia” or “cytokines and antipsychotics” as the search terms, followed by a subsequent one using “schizophrenia childhood trauma” and “schizophrenia microbiome”. In addition, we performed a manual search of the references of articles selected for inclusion in this paper. Only full-text articles published in the English language were included.

## 3. Cytokines and Schizophrenia

Presented in Table 1 are the results of meta-analyses concerning alterations in peripheral (serum or plasma) cytokine levels in FEAN and FEP populations, and Table 2 shows those in ARCh, SCh and CHR/UHR populations. Table 3 presents CSF cytokine levels in patients with schizophrenia. Based on the available meta-analytical data, it is hypothesized that there is a certain disturbance in the balance between pro-inflammatory cytokines, such as interleukin-6 (IL-6) or IL-1β, and anti-inflammatory cytokines such as interleukin 10 (IL-10) [6,29]. This hypothesis is supported by the protective effect of Th2 cytokines and anti-inflammatory cytokines, whose elevated prenatal levels in the maternal blood may reduce the risk of schizophrenia in the offspring [30,31]. Additionally, a meta-analysis by Zhang et al. suggested a particularly large influence of cytokine imbalance on the risk of schizophrenia in the offspring in the early stages of pregnancy [31]. Elevated levels of proinflammatory cytokines may cause the overactivation of astrocytes and microglia, and the presynaptic stimulation of dopaminergic receptors in the midbrain [32]. They are also known to affect kynurenine pathway regulation and disturb glutamatergic transmission [33].

A summary of the general effect of antipsychotics on cytokine levels is available in Table 4, while results stratified by population can be found in Table 5.

### 3.1. Interleukin-1β

Considered one of the main pro-inflammatory cytokines, interleukin-1β (IL-1β) is secreted primarily by monocytes, macrophages, microglia and lymphocytes in response to lipopolysaccharide (LPS), other cytokines and complement fragments [34,35]. It is known to activate the expression of numerous genes, including cytokine genes, and increase the secretion of adrenocorticotropin (ACTH) [34], while changes in its levels are reported to disturb neuronal migration [36].

In the case of FEAN patients, meta-analytical data seem to yield somewhat inconsistent results with regard to the peripheral levels of IL-1β as compared to HC, with three meta-analyses by Miller et al., Upthegrove et al. and Goldsmith et al. indicating its increased levels in this patient population [12,13,14], and two more recent ones by Pillinger et al. and Çakici et al. failing to replicate these results [16,37]. In ARCh patients, there are reports of elevated peripheral levels of IL-1β compared to HC [14], while in the SCh population, they seem to be increased [14] or unaltered [12]. There is evidence of reduced IL-1β levels in the CHR population as compared to HC [38], but a more recent meta-analysis by Misiak et al., including a larger sample of CHR and UHR patients, did not confirm such findings [39]. As regards chronic EOP patients, no significant differences in IL-1β levels were found compared to HC [40]. The results of meta-analyses concerning IL-1β CSF levels are also vastly inconclusive, showing their decreased [12], increased [41] or unaltered [42,43] values compared to HC, although some observations are based on rather small research samples [12].

Interleukin-1 receptor antagonist (IL-1RA) peripheral levels also appear to be elevated in both FEAN [14] and ARCh populations [12,14]. Since IL-1RA is known to reduce the pro-inflammatory effect of IL-1β, its higher levels may result from elevated IL-1β levels [12,14,44].

IL-1β peripheral levels correlate with positive and negative symptom severity and the overall psychopathological presentation [45,46,47]. A correlation has also been demonstrated between elevated IL-1RA levels and cognitive deficits [45].

Where no stratification of patients is applied, either by population or by specific medications used, antipsychotics are reported to reduce IL-1β levels in the blood [12,14,48,49]. However, one meta-analysis by Capuzzi et al. showed unaltered levels of IL-1β in the blood of FEAN patients in response to antipsychotics [50], while another by Romeo et al., based on a larger sample, showed its decreased levels [48]. The latter effect of antipsychotics was also reported in FEP patients [51]. Antipsychotics seem to also reduce IL-1β peripheral levels in the ARCh population [48]. When stratifying by specific antipsychotics, decreased IL-1β peripheral levels were reported in response to treatment with risperidone [48].

### 3.2. Interleukin-2

IL-2 is produced upon the stimulation of CD28, mainly by T-helper type 1 (Th1) and cytotoxic T (Tc) lymphocytes. Its anti-inflammatory effect follows the initial pro-inflammatory action. What is more, IL-2 induces the secretion of IL-6, interferon γ (IFN-γ) and other inflammatory mediators [34].

There is no evidence of altered IL-2 peripheral or CSF levels in any patient population, or differences between ARCh and SCh patients versus HC [12,14,16,42,43]. In turn, the levels of soluble IL-2 receptor (sIL-2R), which reduces the biological activity of IL-2 by binding it and thus preventing its anti-inflammatory effect, are elevated in the FEAN group compared to HC [12,13,14]. As regards the ARCh population, there are reports of it being either unchanged, in a meta-analysis by Miller et al. [12],; or elevated, in a meta-analysis by Goldsmith et al. [14],; but what is worth noting is that in the former work, the authors considered a much smaller sample. Similar results are observed in SCh patients, although in this case the differences in the size of the research samples were less pronounced [12,14]. Interestingly, there is evidence of reduced levels of sIL-2R in the cerebrospinal fluid compared to HC [41].

A negative correlation was found between peripheral IL-2 levels, negative symptom severity and cognitive deficits [45]. In addition, patients with elevated levels tended to improve more slowly in response to treatment [45]. What is more, there is a positive correlation between sIL-2R levels and the overall psychopathological presentation [46,47].

Where stratification by patient population or specific medications used was not applied, no links between antipsychotics administration and peripheral IL-2 levels were observed [12,14,48,49,50]. A meta-analysis by Romeo et al. showed unaltered levels of IL-2 in the blood of FEAN patients in response to antipsychotics [48], while another by Capuzzi et al., which included a larger sample, showed reduced levels [50]. However, a meta-analysis of IL-2 levels in FEP patients by Marcinowicz et al. based on the largest sample showed no antipsychotic effect [51]. There is no proven effect of antipsychotics on IL-2 levels in the blood of the ARCh population [48]. Stratification by administered pharmacotherapy found reduced IL-2 levels in response to risperidone, olanzapine and haloperidol, but not to quetiapine [48]. However, such an effect may be temporary [45]. There are consistent reports of elevated peripheral levels of sIL-2R in patients with schizophrenia treated with antipsychotics where no stratification is applied [12,14,48,49], although such an effect has not been observed in AChR patients [48]. Interestingly, elevated levels of sIL-2R were found in response to antipsychotics in both drug-resistant patients and those treated with clozapine, which is the key drug used in this population [48].

### 3.3. Interleukin-4

A pro-inflammatory cytokine, IL-4, is produced by activated Th lymphocytes (mainly Th2 helper lymphocytes), NKT (natural killer T-cell) lymphocytes, mast cells and basophils. Among others, its role is to promote the differentiation of Th into Th2 lymphocytes, and it also increases their cytotoxicity [34]. It is particularly important that it is also known to affect macrophages and microglial cells, and may have a neuroprotective effect by reducing their ability to induce oxidative stress [40]. In addition, it also plays a role in cognitive processes [11].

Although Goldsmith et al. showed reduced levels of IL-4 in FEAN patients [14], three other meta-analyses, including two based on much larger samples, showed its unaltered levels compared to HCs [13,16,37]. In addition, Goldsmith et al. indicated decreased IL-4 peripheral levels in ARCh compared to HCs, and theirs was the only meta-analysis of IL-4 blood levels in this patient population [14]. No alterations of IL-4 levels were found in the blood of Sch [14] or CHR populations [38]. In addition, decreased IL-4 levels were observed in the chronic EOP population compared to adult AChR and HC, which may indicate that reduced peripheral levels of IL-4 are typical only of AChR patients with early-onset psychosis and comorbid minor neurological disorders [40].

A positive correlation was found between IL-4 peripheral concentration and negative symptom severity, as well as the incidence of depressive symptoms [45].

Although one meta-analysis by Goldsmith et al. showed reduced peripheral levels of IL-4 in response to antipsychotics without stratification by patient population or specific medications [14], two others by Romeo et al. and Tourjman et al. did not confirm such an effect of pharmacotherapy [48,49]. In contrast, Romeo et al. showed decreased peripheral levels of IL-4 in FEAN patients treated with antipsychotics, but not in the ARCh population [48]. Such an effect of antipsychotics in FEP patients is also confirmed by the meta-analysis by Marcinowicz et al. [51]. Stratification by applied pharmacotherapy provides evidence of reduced peripheral IL-4 levels after treatment with risperidone, but not with olanzapine, aripiprazole or clozapine [48].

### 3.4. Interleukin-6

IL-6 is another major pro-inflammatory cytokine produced by macrophages, monocytes, and microglia. Its secretion is induced by IL-1β, interferons, tumor necrosis factor-α (TNF-α), lipopolysaccharide, and viral infections [34]. IL-6 increases the synthesis of acute-phase proteins, including C-reactive protein (CRP), which may affect the permeability of the BBB and the proliferation of microglia [34,38]. Changes in IL-6 levels may disrupt neurogenesis and reduce glutamate reuptake [30]. Soluble IL-6 receptor (sIL-6R) may bind to this cytokine, further increasing its biological activity [14].

IL-6 is one of the most extensively investigated cytokines in schizophrenia research, and with the exception of the meta-analysis by Miller et al., which reported its unaltered levels in SCh patients [12], there are consistent reports of elevated peripheral and CSF levels compared to HC in all patient populations [13,14,41,42,43], including chronic EOP, CHR and UHR patients [38,39,40]. Additionally, evidence suggests a significantly reduced variability in its levels compared to HC, which may indicate that elevated levels of IL-6 is a key element of the schizophrenia immunophenotype [37].

There is a positive correlation between the concentration of IL-6 in the blood and negative and positive symptoms, as well as the overall psychopathological presentation and cognitive deficits [45,46,47]. Interestingly, the meta-analysis by Miller et al. also showed a negative correlation of IL-6 CSF levels with schizophrenia symptom severity [12].

Two meta-analyses by Miller et al. and Goldsmith et al. showed reduced peripheral levels of IL-6 in response to antipsychotics without stratification by specific medications or patient populations [12,14], but two others by Romeo et al. and Tourjman et al. showed its unaltered levels [48,49]. Considering only FEAN patients, metanalyses by Capuzzi et al. and Romeo et al. consistently show reduced IL-6 peripheral levels in response to antipsychotics [48,50]. Similar findings have been reported by Marcinowicz et al., including in FEP patients [51]. The effects of antipsychotics in the ARCh population seem to be similar, unlike in SCh patients, whose IL-6 peripheral levels seem to remain unaffected by antipsychotic treatment [48]. Conversely, IL-6 peripheral levels in treatment-resistant patients increase instead of decreasing [48]. Reduced IL-6 levels are observed only in the case of treatment with risperidone, but not olanzapine, quetiapine, aripiprazole, clozapine or haloperidol [48,52]. Interestingly, IL-6 peripheral levels also appear to increase with the duration of the disease [45]. Treatment with antipsychotics leads to reduced peripheral levels of sIL-6R in the ARCh population, but not in FEAN or drug-resistant patients [48].

### 3.5. Interleukin-8

Secreted primarily in response to an antigen by macrophage, T-lymphocytes, neutrophils and other cells, IL-8 is also the most potent chemokine in humans [34]. This pro-inflammatory cytokine intensifies the migration of neutrophils, T lymphocytes and monocytes, whose enzymes produce oxygen-free radicals and thus increase oxidative stress, which may result in a loss of neurons [12,34].

Research on IL-8 in pregnant women suggests a correlation between its elevated peripheral levels and psychosis in the adult offspring [53]. Except for one meta-analysis by Pillinger et al., which showed no alterations in IL-8 peripheral levels compared to HC [37], research consistently suggests its elevated peripheral levels in FEAN [12,16] and ARCh patients [12,14,41] but not in medicated FEP populations [54]. In turn, no alterations are found in CHR and UHR populations [38,39]. None of the available meta-analyses present evidence of its peripheral levels in SCh.

The IL-8 peripheral concentration shows a positive correlation with negative symptom severity and the overall psychopathological presentation [45]. Patients with elevated IL-8 levels improve more slowly during treatment, and have a poorer prognosis for negative symptoms [45]. IL-8 levels appear to increase with disease duration [45].

Where no stratification is considered, either by patient population or administered pharmacological treatment, antipsychotic medications do not seem to affect peripheral levels of IL-8 [48]. Likewise, such an effect is not observed when considering only FEAN patients or only those treated with risperidone [48].

### 3.6. Interleukin-10

IL-10 is one of the primary anti-inflammatory cytokines secreted mostly by activated regulatory T (Treg), Th2 lymphocytes and regulatory B lymphocytes [55]. It reduces the production of reactive oxygen species and contributes to reduced oxidative stress [56]. It also reduces the secretion of IFN-γ and IL-2 by Th1 lymphocytes and pro-inflammatory cytokines by macrophages [34].

Although one meta-analysis by Goldsmith et al. showed elevated levels of IL-10 in the blood of FEAN patients [14], two later ones by Pillinger et al. and Çakici et al. involving larger samples indicated its unchanged levels in this cohort relative to HC [16,37]. Interestingly, ARCh patients seem to have reduced peripheral IL-10 levels versus HC, but such observations are reported in only two studies with relatively small samples [12,14]. Only one meta-analysis by Goldsmith et al. considered IL-10 peripheral levels in SCh patients, demonstrating their unaltered peripheral levels [14]. Similarly, peripheral levels of IL-10 also appear to be unchanged relative to HC in the CHR and UHR populations [38,39]. Interestingly, reduced IL-10 peripheral levels relative to both other adult AChRs and HCs were demonstrated in AChR patients with early-onset psychosis [40], which is inconsistent with the results reported in other works [12,14].

A positive correlation has been found between peripheral levels of IL-10 and negative symptom severity, general psychopathological presentation, attention deficits and incidence of aggressive behaviors, and a negative correlation with cognitive deficits [45].

Available meta-analyses in which no stratification by treatment or patient population has been applied suggest no effect of antipsychotic medications on peripheral IL-10 levels [48,49]. Similar observations were made for the FEAN and ARCh populations [48]. However, in their meta-analysis employing a much larger sample, Marcinowicz et al. showed decreasing peripheral levels of IL-10 in response to antipsychotic treatment [51]. Stratification by treatment revealed that risperidone lowers, aripiprazole elevates, while quetiapine has no effect on IL-10 levels [48].

### 3.7. Interleukin-12

Secreted mainly by macrophages and dendritic cells in response to components of the bacterial cell wall, IL-12 stimulates proliferation, and activates and increases the cytotoxicity of NK and T cells, promoting the differentiation of the latter into Th1. It is also known to induce the secretion of IFN-γ and TNF-α, and has a synergistic effect with IL-18 [34,57].

Although in their meta-analysis Miller et al. showed increased IL-12 peripheral levels in FEAN compared to HC [12], further meta-analyses by Goldsmith et al. and Çakici et al. based on much larger samples found no such alterations [14,16]. Only the meta-analysis by Miller et al. investigated variations in IL-12 peripheral levels in a SCh population, failing to show any differences in relation to HC [12]. Likewise, there was no evidence of alterations in IL-12 CSF levels [12]. Reduced IL-12 levels were reported in chronic EOP patients, but not in CHR [38,39,40].

IL-12 peripheral levels appear to correlate with cognitive deficits [45].

Meta-analyses of the effect of antipsychotics on IL-12 peripheral levels where no stratification by population or administered pharmacotherapy is applied seem to yield inconsistent results, suggesting their elevated [12,14,49] or unaltered values [48]. In addition, in their meta-analysis including the largest sample, Romeo et al. described no changes in IL-12 peripheral levels in ARCh patients, and their elevated values in those treated with risperidone [48].

### 3.8. Interleukin-17

IL-17 is secreted by helper lymphocytes 17 (Th17) and stimulates, among others, macrophages and microglia to secrete proinflammatory cytokines [34].

In their meta-analyses both Goldsmith et al. and Fang et al. showed no alterations in IL-17 peripheral levels in FEAN patients compared to HC [14,15]. Nevertheless, the most recent meta-analysis by Pillinger et al., based on the largest sample, found its elevated peripheral levels in the FEAN population [37].

Studies indicate a positive correlation of IL-17 with positive symptom severity and overall psychopathological presentation, as well as incidence of aggressive behaviors, and a negative correlation with negative symptoms [45,46,47].

To date, no effect of antipsychotics on peripheral IL-17 levels has been demonstrated [12,48,50,51].

### 3.9. Interleukin-18

Interleukin 18 (IL-18) is a pro-inflammatory cytokine produced by macrophages and dendritic cells and astrocytes, as well as in several types of epithelial cells [34]. By interacting with IL-12 or IL-15, it induces the secretion of IFN-γ by Tc lymphocytes and NK cells, and stimulates the differentiation of Th1 lymphocytes [57], while where no such interactions occur, it mediates the differentiation of T cells into Th2 cells [34,57].

To date, only one meta-analysis by Goldsmith et al. considered IL-18 peripheral levels in psychosis, including only the FEAN population and demonstrating its unaltered levels in this cohort compared to HCs [14]. There are some reports of elevated [58] and unchanged [59] IL-18 levels in the EOP population, as well as in the ARCh patients [60]. The IL-18-binding protein (IL-18BP), whose main role is probably to blunt IL-18 and Th1 activity in order to prevent autoimmune response, may also be of importance in schizophrenia [57]. An elevated ratio of IL-18 to IL-18BP has been demonstrated in EOP, thus suggesting the increased biological activity of IL-18 in this patient population [59].

Peripheral levels of IL-18 seem to decrease in response to antipsychotics in the AChR population [60].

A correlation between peripheral levels of IL-18 and the severity of depressive symptoms was found in EOP, which is probably related to the increased cortisol level [59].

### 3.10. Tumor Necrosis Factor α

Produced predominantly by macrophages (18), for which it is a chemotactic factor, TNF-α increases their cytotoxicity and the ability of the entire immune system to induce oxidative stress [56]. Many other cytokines, including IL-1, IL-6, and IFN-γ, are secreted in response to TNF-α [34]. Produced also by microglia, TNF-α increases the mortality of hippocampal stem cells, affects neuroplasticity, and reduces neurogenesis [29]. Soluble TNF receptors (sTNF-R2, sTNF-R) have proven neuroprotective and immunosuppressive effects [61].

Available meta-analyses consistently report elevated peripheral TNF-α levels in FEAN [12,13,14,16,37] and AChR [12,14]. Conversely, although one meta-analysis by Miller et al. showed unaltered TNF-α levels in the SCh population [12], another one by Goldsmith et al., based on a larger sample, suggested its elevated peripheral levels [14]. Recent evidence appears to yield even more inconsistencies, demonstrating reduced TNF-α peripheral levels relative to controls in both EOP and ARCh patients [38], and no changes in the CHR population [39,40].

There is a positive correlation between peripheral TNF-α levels and negative symptom severity, as well as a negative correlation with positive symptoms in all populations, general psychopathology in AChR, and cognitive deficits [45].

Meta-analytical data, where no stratification by patient has been performed, suggest that antipsychotics do not affect the peripheral levels of TNF-α [12,14,48,49]. Authors are also consistent in reporting a lack of such effects in the FEAN population [48,50], and reduced TNF-α levels in response to antipsychotic medication in the ARCh group [48]. Likewise, Marcinowicz et al. also demonstrated decreased peripheral levels of TNF-α in the patients treated with antipsychotics [51]. Stratification by treatment suggests that olanzapine can lower the levels of TNF-α in the blood, but no similar effect is found for risperidone, quetiapine, aripiprazole or clozapine [48]. Antipsychotics also elevate peripheral levels of sTNF-R1 and sTNR-R2. In the case of the latter, the effect is reported without considering any stratification of patients, and in the case of both receptors, drug-resistant patients or those treated with clozapine [48].

### 3.11. Interferon γ

Interferon γ (IFN-γ) is produced by T lymphocytes, NK and NKT cells activated by the action of other cytokines (IL-2, IL-12, IL-15, IL-18, IL-21) (18). IFN-γ is involved in stimulating the antiviral response and is an activator of macrophages, increasing their cytotoxicity. Moreover, it induces the secretion of IL-6, IL-15 and TNF-α [34].

Meta-analyses of IFN-γ blood levels in the FEAN population yield inconsistent results, demonstrating their elevated [12,14,37] or unaltered values compared to HCs [13,16]. Elevated IFN-γ levels compared to HCs are reported in AChR patients [12,14]. As regards the SCh population, meta-analytical data are inconsistent, suggesting unaltered [12] or reduced IFN-γ peripheral levels compared to HCs [14]. No IFN-γ alterations of any type were found in the CHR or UHR populations [38,39]. IFN-γ peripheral levels correlate with positive symptom severity and general psychopathology in the FEAN population [45,46,47]. More pronounced negative symptoms are observed in patients with higher peripheral levels of IFN-γ [46,47]

Two meta-analyses wherein no stratification by population or treatment was performed found no changes in IFN-γ peripheral levels in schizophrenia patients compared to HCs [12,14], but two others based on larger samples suggested their reduced values in the patient population [48,49]. Both Capuzzi et al. and Romeo et al., detailing the effects of antipsychotics on the FEAN cohort, have shown that IFN-γ levels do not change in response to treatment [48,50]. However, they do seem to decrease in ARCh patients [14] and the FEP population [51]. Where stratification by treatment was applied, a decrease in peripheral IFN-γ levels was demonstrated in response to therapy with olanzapine, but not risperidone, quetiapine or aripiprazole [48].

### 3.12. Transforming Growth Factor β

Transforming growth factor β (TGF-β) is an anti-inflammatory cytokine produced by macrophages, neutrophils, platelets and lymphocytes. It inhibits the proliferation of B and T lymphocytes, as well as NK cells. Instead, it increases Treg and Th17 lymphocytes and reduces the secretion of numerous cytokines [34].

Although three meta-analyses suggest elevated TGF-β peripheral levels in FEAN patients compared to HCs [12,14,37], another one demonstrates their unaltered values in the same patient group [16]. Elevated TGF-β levels were found also in ARCh patients [12,14], but no alterations in their values were reported in the SCh population [12].

General psychopathology is reported to correlate with TGF-β levels [45,47]. Furthermore, more severe negative symptoms are observed in patients with higher peripheral levels of TGF-β [46]. 

Although one meta-analysis wherein no stratification by patient population was applied showed reduced peripheral levels of TGF-β after the administration of antipsychotics [12], three subsequent ones, based on larger samples, did not confirm such an effect [14,48,49]. Similarly, considering only the ARCh population, the meta-analysis by Romeo et al. showed no alterations in peripheral levels of TGF-β in response to antipsychotic treatment [48].

### 3.13. Chemokines

Chemokines are a family of cytokines that are mainly chemotactic factors for cells of the immune system, three of which appear to play a role in schizophrenia [54]. Monocyte chemoattractant protein 1 (MCP-1, CCL2) is a chemokine induced mostly by pro-inflammatory cytokines (e.g., IL-1, IL-4, TNF-α, IFN-γ), but also by growth factors, lipopolysaccharides, reactive oxygen species and the immune complex [62]. MCP-1 interaction with CC chemokine receptor 2 leads to the activation of specific intracellular pathways, recruiting monocytes into sites of inflammation [62]. Moreover, MCP-1 plays an important role in the differentiation of T helper (Th) lymphocytes toward a Th1 or a Th2 phenotype [62]. Macrophage inflammatory protein 1β (MIP-1β, CCL4), which binds to CCR5, induces lymphocyte migration and adhesion, as well as the activation of ROS production [63,64]. In turn, eotaxin (CCL11) is mostly recognized as an eosinophile-specific chemokine related to complement component 3 [65]. The receptor for eotaxin is also expressed on basophiles, mast cells and Th2 lymphocytes, impacting their immune response [66].

The relationship of schizophrenia with peripheral chemokine levels has not been studied as extensively as the effect of non-chemokine cytokines on its course and development [54]. In addition to the previously reported IL-8 alterations, Frydecka et al. showed elevated peripheral levels of MCP-1, MIP-1β and eotaxin, where no stratification by patient group was applied, as well as elevated levels of MCP-1 in FEP, and MCP-1 and eotaxin in ARCh [54]. Interestingly, Misiak et al. also indicated an elevated concentration of MCP-1 in the blood of UHR [39].

## 4. Immunogenetics of Cytokine Alternations in Schizophrenia

The role of immunological dysfunction in the etiopathogenesis of schizophrenia is also demonstrated by its association with disorders of known autoimmunological underpinnings [67]. Schizophrenia has been shown to be associated with major histocompatibility complex A gene (MHC-A) polymorphisms [68], and with rheumatoid arthritis through major histocompatibility complex class II (MHC-II) or DRB1β chain gene (HLA-DRB1) [7].

Schizophrenia has also been shown to be associated with polymorphisms of numerous genes responsible for the synthesis of such cytokines as IL1A, IL1B, IL10 and IL6, which are genes for, respectively, IL-1α, IL-1β, IL-10 and IL-6 [67,68]. The polymorphism of the IL6 (-174G/C) gene does not elevate the level of IL-6 in the serum, but it has been shown to be associated with the occurrence and severity of positive symptoms in the course of schizophrenia [69]. Polymorphisms of TGFB1, which encodes TGF-β, along with its additional overexpression [70] were also associated with schizophrenia [71]. Quite remarkably, polymorphisms of the TNFR2 gene encoding the TNF-α receptor, depending on the variant, may be associated with an increased risk of schizophrenia, or may have a protective effect [72].

## 5. The Role of Early Childhood Trauma

Early childhood trauma has been associated with elevated peripheral levels of IL-6 and a faster elevation in IL-6 and IL-1β levels with age in healthy adults, which, in the case of IL-6, also seems to have an impact on neurophysiology, but not IQ [73,74,75,76]. Experiencing any type of trauma in early childhood seems to lead to elevated peripheral levels of TNF-α and IL-6 in adults, while being a victim of sexual abuse only elevates peripheral levels of TNF-α in adulthood [77]. The nature of the relationship between early childhood trauma and cytokine levels also seems to depend on a number of factors, such as the nature of the trauma, applied diagnostic categories, age, menopause, and gender [77,78,79].

A study by Dennison et al. showed that only those schizophrenia patients who experienced childhood trauma had elevated levels of TNF-α and IL-6, while those who did not report such experience had levels of these cytokines similar to the control group [80]. Another study by Di Nicola et al. showed that although all FEP patients had elevated cytokine levels relative to the control group, those who experienced childhood trauma had higher serum TNF-α levels than those who did not [81]. The effect of childhood trauma on the elevated expression of IL-6 seems to be greater in schizophrenic patients than in the healthy controls, although such observations were based on small control and research groups [82]. A recent study by Corsi-Zuealli et al. conducted on a much larger sample of FEAN patients and HC, including healthy siblings of patients, showed that the peripheral levels of TGF-β in participants who had experienced physical abuse in childhood were significantly elevated in patients and their siblings compared to the controls. Moreover, patients who experienced physical abuse had higher levels of TGF-β than those who did not. This study suggests that elevated peripheral levels of TGF-β, but not IL-6, IL-1β, TNF-α, IFN-γ or IL-10, may not only predispose one to the development of schizophrenia, but may also be a consequence of childhood trauma [83]. Childhood trauma also seems to play a mediating role between elevated TNF-α levels and the changes in gray matter visible in MRI studies in patients with schizophrenia [84].

## 6. Gut Microbiome Dysbiosis in Schizophrenia

Gut microbiome dysbiosis affects behavior as well as the functioning and maturation of microglia [85,86]. Dysbiosis may also influence the activity of astrocytes with the participation of type I interferons and tryptophan metabolites [87]. Disturbances in the composition of the intestinal microbiome are associated with an increased permeability of the intestinal epithelium for bacteria, their antigens, and pathogen-associated molecular patterns (PAMPs), e.g., LPS. Greater permeability, in turn, correlates with elevated peripheral cortisol levels and could lead to the activation of the immune system related to the secretion of pro-inflammatory cytokines, activation of the HPA axis and the creation of a positive feedback loop [88,89,90,91].

In schizophrenia, there are differences in the composition of the microbiome compared to healthy controls, both in the oropharynx and in the intestines [26,27,92]. Gut microbiome biodiversity is greater in patients with schizophrenia, and shows a negative correlation with the number of CD8+ memory T cells in the blood [28]. The population size of individual bacterial species in the microbiome also correlates with symptom severity, and disturbances in its composition may be a marker of response to pharmacotherapy in FEP [93]. Moreover, an altered composition of gut microbiota persists despite treatment with olanzapine, which does not appear to have a significant effect on it [94]. Although there is no evidence of a beneficial effect of probiotic supplementation on schizophrenia symptom severity, it seems possible to use microbiome tests as an auxiliary tool in the diagnosis of the disease [27,95]. Further research is required to establish whether and to what extent dysbiosis may be responsible for the disturbances in the cytokine system present in patients with schizophrenia.

## 7. Association of Alterations in the Cytokine Network with Neuroimaging

Changes in the structure of the brain in patients with diagnosed schizophrenia are well reported in the literature [96]. Reduced gray matter and enlarged ventricles are described in FEP patients [97]. Additionally, within the white matter, there are also many aberrations that suggest impaired connectivity between different areas of the brain [98].

There is a growing body of evidence for a relationship between alterations in cytokine levels and the structure of gray and white matter of the brain in schizophrenia. Cytokines that can easily penetrate the blood–brain barrier induce reactions that can cause a cascading inflammatory response within the central nervous system [11], which leads to the activation of microglia and a reduction in the number of astrocytes, neurotoxicity, abnormal synaptic pruning or nerve cell apoptosis [11,99].

Elevated levels of pro-inflammatory cytokines (IL-6 and TNF-α) correlate with altered gray matter [84]. The level of IL-6 turns out to be significantly correlated with cortical thickness in the left pars opercularis, right pars triangularis, left superior temporal gyrus, and right middle temporal gyrus [100]. Other reports show a negative correlation between elevated levels of IL-1β mRNA (peripherally tested) and a reduced volume of Broca’s area and verbal fluency in schizophrenia [101]. On the other hand, Hoseth et al. found no relationship between the structure or the volume of the hippocampus and plasma cytokine levels [102]. There is evidence that fetal exposure to elevated levels of IL-8 in maternal serum leads to reduced volumes of posterior cingulate and left entorhinal cortex, and increased volumes of ventricular cerebrospinal fluid, in patients with schizophrenia [103]. Neuroinflammation may also be associated with disorders of the white matter, and thus be the cause underlying altered brain connectivity [104]. It can lead to axonal degeneration, destruction of myelin and reduction of the number of oligodendrocytes. Changes in water anisotropy within the white matter fibers (which may be associated with altered white matter quality and lead to disturbances in connections between different areas of the brain [105]) may correlate with IL-6 levels in patients with schizophrenia [106]. As reported by Di Biase, peripheral levels of IL-6 and TNF-α correlate with the amount of free water (FW). The increase in FW is sometimes associated with inflammation, and is found within white matter in patients with schizophrenia [107]. However, whether this type of neuroinflammation may lead to direct changes in white matter remains unresolved [8]. All in all, the relationship between altered cytokine levels and structural changes in the brain is still unclear.

## 8. Glial Dysfunction

The dysfunction of glial cells, which are the second component of nervous tissue besides neurons, is proposed as one of the key elements of the etiopathogenesis of schizophrenia, and at least some of the abnormalities affecting them may be mediated by cytokines [108]. Both autopsy examinations of brains [109] and positron emission tomography (PET) using the TSPO marker (18 kDa translocator protein) indicate the excessive activation of microglia, myeloid immune cells of the nervous system, in schizophrenia [110]. As mentioned, the pathological activation of microglia induced by pro-inflammatory cytokines could be associated with the abnormal pruning of neurons, as well as with the increased mortality of astrocytes, oligodendrocytes, neurons, and structural changes in both white and gray matter, demonstrated by neuroimaging studies in patients with schizophrenia [104,109]. Abnormal maturation and microglia activation could be associated with elevated levels of IL-6 and TGF-β in the course of schizophrenia [14,16,111,112].

However, cytokines may also have a detrimental effect directly on astroglial metabolism, for example, on the regulation of the kynurenine pathway, which is particularly active in this cell population [113]. The main regulatory enzyme of this pathway, indoleamine-2,3-oxidase (IDO), is induced by IFN-γ, the effect of which may be counteracted by IL-4 and IL-10 and enhanced by IL-1β [114]. Changes in the activity of IDO and other enzymes of the kynurenine pathway caused by disturbances in the cytokine network lead to an increased accumulation of kynurenic acid, the levels of which are increased in the CSF, brain tissue and blood of patients with schizophrenia [33,114,115]. Kynurenic acid is an NMDAR antagonist, and although physiologically it may protect against glutamate excitotoxicity, its excess may lead to NMDAR hypofunctionality, contributing to further increases in oxidative stress, the intensification of neuroinflammation, as well as disorders of the glutamine–glutamate cycle and the improper regulation of glutamatergic metabolism, ultimately exacerbating exotoxicity [6,116,117]. Parvalbumin interneurons (PVI), which play a key role in the regulation of dopaminergic pathways, whose dysfunction has a proven role in the development of schizophrenia symptoms, could be particularly sensitive to the consequences of such astrocyte dysfunction [6,117]. In addition, IL-1β increases the expression of FOXP3 and CCL20 genes, the roles of which are also postulated in the etiopathogenesis of schizophrenia, more in patient astrocytes than in HC astrocytes. [118]. Altered patterns of gene expression and astrocyte differentiation could also contribute to disorders of oligodendrocyte maturation and abnormal myelination [108].

## 9. The Role of Nuclear Factor-κB and Human Endogenous Retroviruses

Nuclear factor-κB (NF-κB), which mediates the increase in the secretion by microglia of such cytokines as IL-1β and IL-18 and is activated by IL-1β, TNF-α, PAMP, adrenal cortex hormones and adrenaline, may play an important role in the potential pathological feedback loop associated with disturbances in the cytokine network in schizophrenia [119]. The increased expression of NF-κB and related receptors and kinases in the cerebral cortex compared to HC is confirmed by autopsy studies of the brains of people diagnosed with schizophrenia [120,121]. Elevated levels of IL-1β and TNF-α, well documented in meta-analytic data, could therefore partially result from or be the cause of increased NF-κB activity [14,16,119]. Other factors influencing the increased activation of NF-κB could also include the well-confirmed increased blood cortisol levels in patients with schizophrenia [122], the indirect dysbiosis of the intestinal microbiome [123], and, with the participation of epigenetic mechanisms, early childhood trauma [124].

Human endogenous retroviruses (HERVs) are a group of viruses that infected germinal cells of mammals many millions of years ago and were permanently integrated into their genome [125]. Under the influence of viral infections, for example, with Epstein–Barr viruses (EBV) and Herpes simplex type 1 (HSV-1), some HERVs may reactivate, the role of which has been proposed, for example, in the etiopathogenesis of multiple sclerosis [126,127]. HSV-1 infections, similarly to other infections during pregnancy and birth in the fall and winter, when such infections are more frequent, are a confirmed risk factor for the development of schizophrenia in the offspring [128]. HSV-1 exposure is also associated with greater cognitive deficits in the course of schizophrenia [129]. It has been suggested that HERVs reactivation may be mediated by NF-κB with the involvement of TNF-α and INF-γ, whose peripheral levels are elevated in schizophrenia [14,130,131]. In turn, the increase in HERVs expression may be associated with both an increase in the expression of pro-inflammatory cytokines and the activation of microglia, thus creating another pathological positive feedback loop [132,133]. Moreover, one of the HERVs, HERV-W, increases the expression of TNF-α and IL-10 in glial cells by inhibiting MyD88s, which is a splice variant of the activator NF-κB (MyD88), which in turn is activated, inter alia, by IL-1β [134,135,136]. It has been proposed that the activation of microglia and peripheral myeloid cells caused by HERVs reactivation could lead to an increase in oxidative stress, and thus damage to astrocytes, disturbance of their function, and abnormal myelination [125].

A study by Karlsson et al. demonstrated HERV-W expression in FEP patients, which is absent in HCs [137]. Similarly, although based on a rather small sample, a post-mortem study by Frank et al. indicated an increased expression of HERV-K10 in the brains of schizophrenia patients [138]. HERV-K methylation in the genetic material obtained from peripheral blood leukocytes is also significantly lower in both FEP and ARCh patients compared to HC [139]. In addition, the increased expressions of HERV-K and HERV-W are also correlated with increased expressions of DISC1, PRODH, BDNF and D3 genes, which are associated with susceptibility to schizophrenia [125,140,141]. Interestingly, the presence of HERV-W antigens in the peripheral blood was also associated, in at least a certain subpopulation of schizophrenic patients, both with elevated peripheral levels of IL-6 and a higher incidence of early childhood trauma resulting from emotional abuse [142].

## 10. Patient Stratification

To date, it has not been established whether cytokine disturbance in schizophrenia occurs in all affected patients, or whether it is rather specific to an “immune” subpopulation thereof. Determining possible patient phenotypes is therefore among the most important research goals. A meta-analysis by Pillinger et al. showed a reduced variability of IL-1β, IL-4, IL-6, IL-8 and TNF-α levels in FEP compared to the control group, which, along with the unimodality of the distributions, could suggest the absence of an immune subgroup of patients and the prevalence of cytokine dysregulation in patients with schizophrenia [37]. Cluster analysis allowed the identification of four subpopulations of FEAN patients with different symptom profiles, with a population with the highest symptom severity characterized by elevated peripheral levels of IL-7, IL-15, IL-17, IFN-γ, TNF-α, soluble intracellular adhesion molecule 1 (sICAM-1) and soluble vascular cell adhesion molecule 1 (sVCAM-1), and the lowest symptom severity population with reduced CXCL12 or elevated IL-8 peripheral levels, compared to the other subpopulations [143].

Due to the heterogeneity of schizophrenia, numerous efforts have been made to distinguish its different subtypes. One such subtype was described in 1988 by Carpenter et al. as deficit syndrome [144], characterized by the presence of negative symptoms (diminished emotional range, restricted affect, diminished sense of purpose, poverty of speech, diminished social drive, curbing of interests), which must be persistent, present for 12 months, and primary (independent of treatment and depressive symptoms, or not due to positive symptoms) [145]. Apart from the characteristic psychopathological presentation, differences in cytokine levels also suggest the presence of a particular schizophrenia subtype. It has been reported that IL-17 elevated peripheral levels compared to HC in deficit schizophrenia [146]. Garcia-Rizo et al. demonstrated higher IL-6 and CRP levels in drug-naive patients with features of deficit syndrome compared to patients with diagnosed schizophrenia [147]. It therefore seems that the peripheral levels of IL-6 and TNF-α may be closely related with the deficit subtype [148].

## 11. Conclusions

In research on schizophrenia, attempts are often made to divide various types of deviations from HC into trait markers that are associated with hereditary and neurodevelopmental factors, and thus susceptibility to the disease, and state markers associated with the disease itself and its symptoms [149]. As available meta-analyses are often inconsistent in distinguishing trait and state markers, we have made an attempt to resolve this issue, using specific criteria. Therefore, we assumed that in order to define an alteration in the peripheral levels of a given cytokine in patients with schizophrenia relative to HC as a trait marker, the following criteria should be met:

(1) Evidence of altered levels of a given cytokine in patients with schizophrenia and in the CHR/UHR population;
(2) Evidence of alterations in both psychosis and remission;
(3) Evidence of alterations in all patient populations.

In the case of discrepancy between meta-analyses, we preferred those based on larger samples for a given cytokine.

Following these criteria, the only cytokine with elevated peripheral levels that can be distinguished as a trait maker based on the results of meta-analyses by Goldsmith et al., Misiak et al. and Çakici et al. is IL-6 [14,16,39]. Although the meta-analysis by Miller et al. yields conflicting results [12], meta-analyses by Goldsmith et al. [14] and Çakici et al. [16],; including much larger patient samples and studies on the CHR/UHR populations, seem to confirm such a proposal [38,39]. It is also supported by larger studies of CSF levels [41,42,43].

Elevated peripheral levels of MCP-1 are also a potential trait marker; however, despite evidence from the FEP population, meta-analytic data on FEAN are lacking [54]. Based on the meta-analysis by Goldsmith et al., trait markers could also be elevated peripheral levels of IL-1β and TNF-α [14]; however, in their case there is no confirmation in the meta-analyses of peripheral levels in the CHR/UHR population [38,39]. Similarly, elevated levels of sIL2-R could be a trait marker of schizophrenia [13,14], but meta-analyses in the CHR/UHR populations did not include them [38,39]. In the case of IL-1β, IL-6 and TNF-α, their potential roles as trait markers of schizophrenia seem to be supported by correlations with the results of neuroimaging studies [84,100,101,102,103,104,105],; and in the case of TNF-α and IL-6, also with the experience of early childhood trauma [80,81].

In order to define an alteration in the peripheral levels of a given cytokine in patients with schizophrenia relative to HC as a state marker, only one of the following criteria should be met:

(1) Evidence of alternated peripheral levels in acute psychosis (FEAN, FEP, ARCh), but not in patients in remission (SCh);
(2) Evidence of inverse or no alterations in peripheral levels in stable chronic (SCh) and acute psychotic (FEAN, FEP ARCh) patients.

Again, in case of discrepancies between meta-analyses, we preferred those based on larger samples for a given cytokine.

According to these criteria and based on the meta-analyses by Miller et al. and Goldsmith et al. [12,14],; state markers of schizophrenia may include elevated peripheral levels of IFN-γ and TGF-β, while lower IFN-γ levels relative to HCs observed in remission [14] may be the result of pharmacotherapy [48]. It is also possible that IL-4 and IL-10 are state markers of ARCh, but not of FEP/FEAN [12,14,37], which seems to be consistent with the different effects of antipsychotics on IL-4 levels in FEAN and ARCh, and with the different effects of individual medications on the levels of IL-10 in the blood [48].

Peripheral levels of IL-1β, IL-6, IL-8, IL-10, IL-17 and IFN-γ show a positive correlation, and peripheral levels IL-2 and TNF-α have a negative correlation, with the intensity of positive symptoms [45,46,47]. Peripheral levels of IL-1β, IL-6, TNF-α, IFN-γ and TGF-β correlate positively, and IL-17 correlates negatively, with the intensity of negative symptoms [45,46,47]. In turn, cognitive impairment seems to correlate with peripheral levels of IL-1β, IL-4, IL-6 and IL-12, and considering only the AChR population, also with peripheral levels of TNF-α [45]. There is also a negative correlation of cognitive dysfunction with the peripheral levels of IL-2 and IL-10; the more frequent occurrence of aggressive behavior is associated with increased levels of IL-10 and IL-17, and the greater incidence of depressive symptoms with increased peripheral levels of IL-4 and IL-18 [45].

Meta-analyses wherein no stratification by specific medications or patient population was applied suggest that the peripheral levels of IL-1β, TGF-β and IFN-γ decreased in response to antipsychotics [12,14,48,49]. It is less clear whether the levels of IL-6 or IL-4 in the blood also decrease, but meta-analyses based on larger samples did not show such changes [12,14,48,49]. This would be consistent with the fact that elevated IL-6 peripheral levels seems to be a trait marker of schizophrenia [14,39]. On the contrary, peripheral levels of sIL-2R and sTNF-R2 elevate with antipsychotic treatment, which is not unequivocal in the case of IL-12 [12,14,48,49]. In the first episode, antipsychotics seem to have a slightly different effect than in others, with decreasing peripheral levels of IL-4, IL-6, and possibly also IL-1β [12,14,48,50]. Despite some contradictory results, meta-analyses based on the largest research groups indicate that in this population, the level of IL-2 in the blood does not change under the influence of medication in the first episode [12,14,48,50]. The results concerning decreasing peripheral levels of IL-10, TNF-α and IFN-γ are less consistent, showing both decreasing and unaltered levels [12,14,48,49,50]. Only one meta-analysis of the influence of antipsychotics on the levels of cytokines by Romeo et al. took into account the population of patients in further episodes, and showed decreasing levels of IL-1β, IL-6, sIL-6R, TNF-α and IFN-γ in this population [48]. In conclusion, the results of the meta-analyses to date seem to suggest that the influence of antipsychotics on cytokine levels may differ during the course of schizophrenia, potentially contributing to the phenomenon of drug resistance, but still, the number of studies including chronic patients is insufficient to confirm this [14,48,49,50,52]. Risperidone seems to have the most notable effect on cytokine levels by lowering the levels of IL-1β, IL-2, IL-4, IL-6 and IL-10, and increasing the levels of IL-12 [48,52]. In addition, olanzapine appears to lower peripheral levels of IL-2, TNF-α and IFN-γ, while clozapine elevates levels of sIL-2R and TNF receptors, and haloperidol reduces levels of IL-2 [48].

Possible causes of altered levels of cytokines in blood and CSF include polymorphisms in their genes [67,68,69]. These polymorphisms could contribute to later sensitivity to the influence of other factors, such as early childhood trauma and HPA axis dysregulation [84],; or disturbances in the composition of the gut microbiota [26,27,28,92].

Currently, there is an insufficient variety of methods for the early diagnosis, prediction and prognosis of schizophrenia that are not based on symptomatology assessment, and a growing body of evidence suggests that cytokine peripheral levels testing could be used to fill this gap [45]. Potential trait markers, such as the previously mentioned elevated peripheral levels of IL-1β, IL-6, MCP-1, TNF-α and sIL2-R, could be helpful in the early recognition of patients at risk of psychosis [12,14,39]. Potential state markers, such as elevated peripheral levels of IFN-γ and TGF-β, could in turn be useful in detecting relapses [12,14]. Elevated IL-6, IL-8, IFN-γ and soluble TNF receptor levels, as well as reduced peripheral levels of IL-2, have been reported as markers of poorer response to treatment with antipsychotics [45]. Elevated IL-7, IL-15, IL-17, IFN-γ and TNF-α levels could be associated with poorer prognosis, while an elevated level of IL-8 with better prognosis [143]. Additionally, IL-6 and TNF-α peripheral levels’ testing could be used to predict future changes in the negative symptom severity in CHR [43]. It has also been proposed to use cytokine level testing to identify the age of onset in schizophrenia patients [40]. IL-4 seems to be the most promising candidate, offering relatively high sensitivity and specificity, and additional measurements of IL-1β, IL-6, IL-10, IL-12 and TNF-α could further enhance the effectiveness of this approach [40].

Disturbances within the cytokine network that occur in the course of schizophrenia can be analyzed in the context of the research domain criteria framework (RDoC). The goal of RDoC is to define mental illnesses based on their biological signatures, not primarily on the clinical picture [150]. The RDoC matrix enables the analysis of disorders in specific domains (e.g., cognitive systems, systems of social processes) and levels (genes, molecules, cells, neurocircuits, physiology, behavior, self-reports), and the systematization of relationships between them [151]. 

While there is evidence of a role of disturbances in the cytokine network in schizophrenia at the gene level [67,68,69] and the level of individual molecules, such as NF-κB and HERVs [133,134,137,140], the abnormal functioning of microglial cells, astrocytes, oligodendrocytes and neurons [6,108,110,113], physiological markers in the form of altered levels of cytokines peripherally and in CSF [14,41], and their correlation with the intensity of symptoms in the classical sense diagnosed using scales and structured interviews [9,45], there are deficiencies in research both on their relationship with the functioning of specific neurocircuits and in the relationships between individual levels in terms of proposed RDoC domains, rather than more traditional categories of psychopathological symptoms, such as positive or negative symptoms.

Considering that disturbances in the cytokine network (1) provide a chance to distinguish HC from schizophrenic patients [9,14,39], (2) allow us to distinguish groups of patients characterized by similar symptom profiles, including those diagnosed with deficit schizophrenia [143,146,147], and (3) illustrate the different effects of individual antipsychotic drugs on the peripheral levels of various cytokines, the knowledge of the profile of disturbances in the cytokine network in a given patient could enable the selection of personalized treatment [48,51]. Designing future research with the RDoC framework in mind could significantly contribute to the development of specific clinical applications and a better understanding of immune disorders in schizophrenia.

Our paper has certain limitations, mainly due to the fact that we have not used any more systematic form of searching for papers relevant to the issue. Therefore, the limitations of our work are typical for a narrative review [152]. Although we have not included all publications related to the topic of cytokine network alterations in schizophrenia, mainly due to their great number and diversity, given that the multitude of papers cited in our narrative review often obtained contradictory results despite the use of protocols for meta-analyses and systematic reviews, our approach can provide a more accessible and holistic source of information on the current state of knowledge for clinicians and researchers less familiar with the topic. Taking into account the potential future applications of knowledge about the alterations in the cytokine network in schizophrenia in clinical practice and the dynamic development of the field, this may be of value for such readers. We would also like to point out that a significant part of the references cited by us are systematic reviews and meta-analyses, often published very recently, which reduces the risk of omitting relevant publications.

In this narrative review, we presented the most important publications on the disturbances in the cytokine system in the course of schizophrenia, and also presented possible relationships with other important fields of research on this disorder, such as the impact of early childhood trauma, disturbances in the gut microbiota, or neuroimaging. A growing body of evidence has shown the significant role of cytokines in the etiopathogenesis of schizophrenia, as well as their possible application in the diagnosis, prognosis and stratification of patients. Additionally, based on the currently available data, we have proposed the most reliable trait and state markers of schizophrenia.

## Figures and Tables

**Table 1:** Summary of meta-analytical data concerning peripheral cytokine levels in first episode psychosis (FEP) and first episode drug naïve (FEAN) patients as compared to healthy controls.

Marker	Miller et al. (2011)		Upthegrove et al. (2014)		Goldsmith et al. (2016)		Fang et al. (2017)		Frydecka et al. (2018) *		Pillinger et al. (2019)		Çakici et al. (2020)	
	Alt.	nS (nP)	Alt.	nS (nP)	Alt.	nS (nP)	Alt.	nS (nP)	Alt.	nS (nP)	Alt.	nS (nP)	Alt.	nS (nP)
**IL-1β**	↑	3 (151)	↑	3 (99)	↑	6 (333)	NA	0 (0)	NA	0 (0	—	(269)	—	9 (298)
**IL-1RA**	NA	0 (0)	NA	0 (0)	↑	2 (194)	NA	0 (0)	NA	0 (0	NA	0 (0)	NA	0 (0)
**IL-2**	—	4 (116)	—	3 (26)	—	5 (140)	NA	0 (0)	NA	0 (0	—	(205)	—	10 (249)
**sIL-2R**	↑	3 (30)	↑	3 (58)	↑	3 (30)	NA	0 (0)	NA	0 (0	NA	(36)	NA	0 (0)
**IL-4**	NA	0 (0)	—	2 (93)	↓	4 (193)	NA	0 (0)	NA	0 (0	—	(320)	—	8 (308)
**IL-6**	↑	4 (117)	↑	5 (181)	↑	11 (506)	NA	0 (0)	NA	0 (0	↑	(652)	↑	14 (540)
**IL-8**	NA	0 (0)	NA	0 (0)	↑	2 (49)	NA	0 (0)	—	3 (99)	—	(96)	↑	6 (123)
**IL-10**	NA	0 (0)	NA	0 (0)	↑	4 (357)	NA	0 (0)	NA	0 (0	—	(415)	—	10 (567)
**IL-12**	↑	2 (78)	NA	0 (0)	—	3 (258)	NA	0 (0)	NA	0 (0	NA	0 (0)	—	2 (15)
**IL-17**	NA	0 (0)	NA	0 (0)	—	2 (157)	—	5 (313)	NA	0 (0	↑	(413)	NA	0 (0)
**IL-18**	NA	0 (0)	NA	0 (0)	—	3 (335)	NA	0 (0)	NA	0 (0	NA	0 (0)	NA	0 (0)
**TNF-α**	↑	4 (200)	↑	3 (99)	↑	9 (587)	NA	0 (0)	NA	0 (0	↑	(488)	↑	11 (376)
**IFN-γ**	↑	2 (48)	—	3 (103)	↑	7 (452)	NA	0 (0)	NA	0 (0	↑	(344)	—	11 (334)
**TGF-β**	↑	2 (81)	NA	0 (0)	↑	3 (169)	NA	0 (0)	NA	0 (0)	↑	(133)	—	2 (98)

Alt.—alteration, nS—number of studies, nP—number of patients, NA—data not available, — not alternated, ↑—elevated levels, ↓—reduced levels. * This metanalysis included non-drug naïve FEP patients.

**Table 2:** Summary of meta-analytical data concerning peripheral cytokine levels in acute relapsed chronic (ARCh), stable chronic (SCh) and clinical high-risk or ultra-high-risk (CHR/UHR) patients as compared to healthy controls.

Marker	Miller et al. (2011)				Goldsmith et al. (2016)				Frydecka et al. (2018)		Park et al. (2019)		Misiak et al. (2021)	
	ARCh	nS (nP)	SCh	nS (nP)	ARCh	nS (nP)	SCh	nS (nP)	ARCh	nS (nP)	CHR	nS (nP)	CHR/UHR	nS (nP)
**IL-1β**	NA	0 (0)	—	3 (127)	↑	3 (131)	↑	4 (330)	NA	0 (0)	↓	2 (14)	—	3
**IL-1RA**	↑	2 (32)	NA	0 (0)	↑	2 (32)	NA	0 (0)	NA	0 (0)	NA	0 (0)	NA	0
**IL-2**	—	2 (43)	—	4 (132)	—	2 (43)	—	6 (193)	NA	0 (0)	NA	0 (0)	NA	0
**sIL-2R**	—	2 (32)	—	3 (90)	↑	3 (58)	↑	3 (116)	NA	0 (0)	NA	0 (0)	NA	0
**IL-4**	NA	0 (0)	NA	0 (0)	↓	4 (169)	—	2 (73)	NA	0 (0)	—	2 (44)	NA	2
**IL-6**	↑	6 (156)	—	5 (164)	↑	9 (279)	↑	12 (711)	NA	0 (0)	↑	5 (81)	↑	7
**IL-8**	↑	2 (46)	NA	0 (0)	↑	2 (46)	NA	0 (0)	↑	12 (696)	—	3 (47)	—	3
**IL-10**	↓	2 (46)	NA	0 (0)	↓	2 (46)	—	4 (118)	NA	0 (0)	—	2 (15)	—	2
**IL-12**	NA	0 (0)	—	3 (104)	NA	0 (0)	NA	0 (0)	NA	0 (0)	—	3 (47)	—	2
**TNF-α**	↑	4 (78)	—	3 (171)	↑	7 (269)	↑	9 (508)	NA	0 (0)	—	2 (44)	—	4
**TNF-β**	NA	0 (0)	NA	0 (0)	NA	0 (0)	NA	0 (0)	NA	0 (0)	—	2 (15)	NA	0
**IFN-γ**	↑	2 (57)	—	2 (62)	↑	4 (162)	↓	4 (198)	NA	0 (0)	—	2 (24)	—	5
**TGF-β**	↑	2 (78)	—	2 (119)	↑	6 (243)	NA	0 (0)	NA	0 (0)	NA	0 (0)	NA	0

nS—number of studies, nP—number of patients, NA—data not available, ——not alternated, ↑—elevated levels, ↓—reduced levels.

**Table 3:** Summary of meta-analytical data concerning cerebrospinal fluid cytokine levels in schizophrenia patients as compared to healthy controls.

Marker	Miller et al. (2011)		Wang et al. (2018)		Gallego et al. (2018)		Orlovska-waast et al. (2019)	
	Alt.	nS (nP)	Alt.	nS (nP)	Alt.	nS (nP)	Alt.	nS (nP)
**IL-1α**	NA	0 (0)	—	2 (70)	NA	0 (0)	—	3 (72)
**IL-1β**	↓	2 (13)	↑	3 (57)	—	4 (56)	—	2 (40)
**IL-2**	—	4 (100)	—	4 (114)	—	4 (121)	—	3 (97)
**sIL-2R**	NA	0 (0)	↓	2 (19)	NA	0 (0)	NA	0 (0)
**IL-6**	—	2 (42)	↑	7 (244)	↑	8 (256)	↑	7 (230)
**IL-8**	NA	0 (0)	↑	3 (112)	↑	4 (105)	↑	3 (95)
**IL-12**	—	2 (40)	NA	0 (0)	NA	0 (0)	NA	0 (0)

Alt.—alteration, nS—number of studies, nP—number of patients, NA—data not available, ——not alternated, ↑—elevated levels, ↓—reduced levels.

**Table 4:** Meta-analytical data concerning the effects of antipsychotics on peripheral cytokine levels without stratification by specific treatment or population.

Marker	Miller et al. (2011)		Tourjman et al. (2011)		Goldsmith et al. (2016)		Romeo et al. (2018)	
	Effect	nS (nP)	Effect	nS (nP)	Effect	nS (nP)	Effect	nS (nP)
**IL-1β**	↓	3 (127)	↓	3 (127)	↓	4 (189)	↓	7 (241)
**IL-1RA**	NA	0 (0)	—	5 (113)	NA	0 (0)	—	6 (131)
**IL-2**	—	4 (132)	—	6 (239)	—	4 (132)	—	10 (311)
**sIL-2R**	↑	3 (90)	↑	8 (165)	↑	3 (90)	↑	11 (263)
**IL-4**	NA	0 (0)	—	2 (119)	↓	2 (186)	—	7 (399)
**IL-6**	↓	4 (164)	—	11 (350)	↓	11 (500)	—	21 (784)
**sIL-6R**	NA	0 (0)	—	4 (84)	NA	0 (0)	—	4 (844)
**IL-8**	NA	0 (0)	NA	0 (0)	NA	0 (0)	—	4 (144)
**IL-10**	NA	0 (0)	—	2 (52)	NA	0 (0)	—	8 (210)
**IL-12**	↑	3 (104)	↑	2 (74)	↑	3 (104)	—	4 (121)
**IL-17**	NA	0 (0)	NA	0 (0)	—	2 (193)	—	3 (248)
**IL-23**	NA	0 (0)	NA	0 (0)	NA	0 (0)	—	2 (68)
**TNF-α**	—	3 (171)	—	9 (320)	—	6 (310)	—	19 (579)
**sTNF-R1**	NA	0 (0)	NA	0 (0)	NA	0 (0)	—	5 (91)
**sTNF-R2**	NA	0 (0)	NA	0 (0)	NA	0 (0)	↑	3 (49)
**IFN-γ**	—	2 (62)	↓	3 (138)	—	4 (265)	↓	8 (380)
**TGF-β**	↓	2 (119)	—	3 (156)	—	4 (286)	—	5 (303)

nS—number of studies, nP—number of patients, —— no effect, ↑—levels increased after therapy, ↓—levels decreased after therapy.

**Table 5:** Meta-analytical data concerning the effect of antipsychotics on peripheral cytokine levels in first episode psychosis (FEP), first episode drug naïve (FEAN), acute relapsed chronic (ARCh), stable chronic (SCh) and clinical high-risk or ultra-high risk (CHR/UHR) patients.

Marker	Capuzzi et al. (2016)		Romeo et al. (2018)								Marcinowicz et al. (2021)	
	FEAN		FEAN		ARCh		SCh		Drug Resistant		FEP	
	Effect	nS (nP)	Effect	nS (nP)	Effect	nS (nP)	Effect	nS (nP)	Effect	nS (nP)	Effect	nS (nP)
**IL-1β**	—	4 (112)	↓	4 (179)	↓	6 (223)	NA	0 (0)	NA	0 (0)	↓	7 (276)
**IL-1RA**	NA	0 (0)	NA	0 (0)	NA	0 (0)	—	2 (41)	—	3 (58)	NA	0 (0)
**IL-2**	↓	2 (69)	—	2 (35)	—	9 (279)	NA	0 (0)	NA	0 (0)	—	4 (145)
**sIL-2R**	NA	0 (0)	NA	0 (0)	—	6 (182)	NA	0 (0)	↑	2 (39)	NA	0 (0)
**IL-4**	NA	0 (0)	↓	3 (167)	—	6 (382)	NA	0 (0)	NA	0 (0)	↓	4 (150)
**IL-6**	↓	4 (253)	↓	5 (226)	↓	14 (643)	—	2 (41)	↑	3 (58)	↓	8 (409)
**sIL-6R**	NA	0 (0)	NA	0 (0)	↓	2 (53)	NA	0 (0)	—	2 (31)	NA	0 (0)
**IL-8**	NA	0 (0)	—	2 (49)	NA	0 (0)	NA	0 (0)	NA	0 (0)	NA	0 (0)
**IL-10**	NA	0 (0)	—	3 (104)	—	6 (173)	NA	0 (0)	NA	0 (0)	↓	3 (150)
**IL-12**	NA	0 (0)	NA	0 (0)	—	3 (68)	NA	0 (0)	NA	0 (0)	NA	0 (0)
**IL-17**	—	2 (157)	—	3 (203)	—	3 (248)	NA	0 (0)	NA	0 (0)	—	3 (203)
**IL-23**	NA	0 (0)	NA	0 (0)	NA	0 (0)	NA	0 (0)	NA	0 (0)	NA	0 (0)
**TNF-α**	—	4 (214)	—	6 (260)	↓	12 (452)	NA	0 (0)	—	3 (51)	↓	7 (328)
**sTNF-R1**	NA	0 (0)	NA	0 (0)	NA	0 (0)	NA	0 (0)	↑	2 (39)	NA	0 (0)
**sTNF-R2**	NA	0 (0)	NA	0 (0)	NA	0 (0)	NA	0 (0)	↑	2 (39)	NA	0 (0)
**IFN-γ**	—	2 (157)	—	3 (172)	↓	7 (363)	NA	0 (0)	NA	0 (0)	↓	5 (243)
**TGF-β**	NA	0 (0)	NA	0 (0)	—	4 (286)	NA	0 (0)	NA	0 (0)	NA	0 (0)

nS—number of studies, nP—number of patients, —— no effect, ↑—levels increased after therapy, ↓—levels decreased after therapy.
